# Age- and Tissue-Specific Expression of Senescence Biomarkers in Mice

**DOI:** 10.3389/fgene.2018.00059

**Published:** 2018-02-23

**Authors:** Adam D. Hudgins, Cagdas Tazearslan, Archana Tare, Yizhou Zhu, Derek Huffman, Yousin Suh

**Affiliations:** ^1^Department of Genetics, Albert Einstein College of Medicine, Bronx, NY, United States; ^2^Department of Molecular Pharmacology, Albert Einstein College of Medicine, Bronx, NY, United States; ^3^Department of Medicine, Albert Einstein College of Medicine, Bronx, NY, United States; ^4^Department of Ophthalmology and Visual Sciences, Albert Einstein College of Medicine, Bronx, NY, United States

**Keywords:** cellular senescence, SASP, aging, gene expression, biomarkers

## Abstract

Cellular senescence is a state of irreversible cellular growth arrest accompanied by distinct changes in gene expression and the acquisition of a complex proinflammatory secretory profile termed the senescence-associated secretory phenotype (SASP). Senescent cells accumulate in aged tissues and contribute to age-related disease in mice. Increasing evidence that selective removal of senescent cells can ameliorate diseases of late life and extend lifespan in mice has given rise to the development of senolytics that target senescent cells as anti-aging therapeutics. To realize the full potential of senolytic medicine, robust biomarkers of senescence must be in place to monitor the *in vivo* appearance of senescent cells with age, as well as their removal by senolytic treatments. Here we investigate the dynamic changes in expression of the molecular hallmarks of senescence, including *p16^Ink4a^*, *p21^Cip1^*, and SASP factors in multiple tissues in mice during aging. We show that expression of these markers is highly variable in age- and tissue-specific manners. Nevertheless, *Mmp12* represents a robust SASP factor that shows consistent age-dependent increases in expression across all tissues analyzed in this study and *p16^Ink4a^* expression is consistently increased with age in most tissues. Likewise, in humans *CDKN2A* (*p16^Ink4a^*) is one of the top genes exhibiting elevated expression in multiple tissues with age as revealed by data analysis of the Genotype-Tissue Expression (GTEx) project. These results support the targeting of *p16^Ink4a^* expressing-cells in senolytic treatments, while emphasizing the need to establish a panel of robust biomarkers of senescence *in vivo* in both mice and humans.

## Introduction

Cellular senescence is an irreversible cellular growth arrest that induces profound phenotypic changes. These changes include alterations to chromatin organization, gene expression, and secretory profiles (Campisi, 2013). The specific secretory profile of senescent cells has been termed the senescence-associated secretory phenotype (SASP), and consists of numerous proinflammatory cytokines, chemokines, growth factors, and proteases (Coppe et al., 2010a). Cellular senescence plays a complex role, both beneficial and deleterious, in biological processes such as embryonic development (Munoz-Espin et al., 2013; Storer et al., 2013), wound healing (Jun and Lau, 2010), tissue regeneration (Krizhanovsky et al., 2008), and tumor suppression (Collado and Serrano, 2010), as well as age-related disorders (Childs et al., 2017). Senescent cells accumulate within aged tissues and at sites of age-related pathology *in vivo*, and potentially contribute to the age-related decline of tissue function by affecting the growth, migration and differentiation of neighboring cells, impacting overall tissue architecture, and promoting chronic inflammation (Ovadya and Krizhanovsky, 2014; van Deursen, 2014). Indeed, studies on both progeroid and naturally aged mice showed that selective elimination of *p16^Ink4a^*-expressing senescent cells increased healthspan and lifespan (Baker et al., 2011, 2016). Thus, the selective elimination of senescent cells (senolytics) or the disruptions of the SASP program have been developed as potential therapeutic strategies against aging. However, while *p16^Ink4a^* expression has been used as a classical senescence biomarker, no biomarker of senescence identified thus far is entirely specific to the senescent state (Sharpless and Sherr, 2015). Thus, due to the lack of robust biomarkers of cellular senescence *in vivo*, the precise extent of senescent cell accumulation in aged animals and the functional outcome of such an accumulation, along with the exact target cells of, and removal by, senolytics, remain unclear. Surprisingly, a systematic multi-tissue *in vivo* study of senescence markers during aging has not been conducted in wild-type animals. As a first step to identify robust senescence biomarkers *in vivo*, the goal of the present study was to determine the mRNA expression profiles of a panel of known molecular hallmarks of senescence in multiple tissues at multiple ages in mice.

## Materials and Methods

### Animals

All animals were housed and treated in accordance with protocols approved by the Institutional Animal Care and Use Committee (IACUC) for animal research at Albert Einstein College of Medicine. All mice were CB6F1 hybrids obtained from the aged-rodent colonies of the National Institute on Aging. Four females of each age group (4, 12, 24, and 30 months) were used for analysis. Animals were euthanized by CO_2_ inhalation and organs were harvested and snap-frozen in liquid nitrogen. Total RNA was harvested from snap-frozen tissue by the TRIzol Plus RNA purification kit (Thermo Fisher Scientific) and treated with DNase I to remove carry-over genomic DNA per manufacturer’s protocol.

### Quantitative Real-Time PCR

**FIGURE 1:**
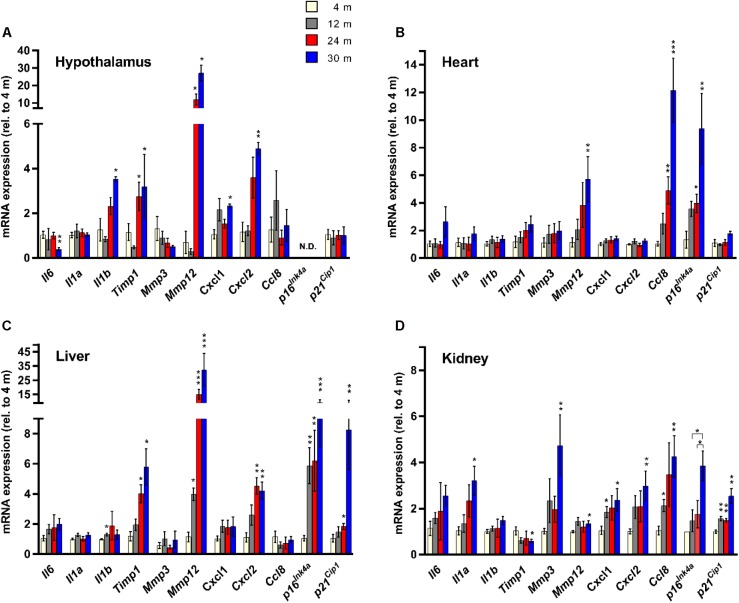
Senescence marker/SASP factor gene expression exhibits age- and tissue-specific patterns. Expression of a panel of SASP factors, and the cyclin-dependent kinase inhibitors *p16^Ink4a^* and *p21^Cip1^*, as determined by RT-qPCR, in hypothalamus **(A)**, heart **(B)**, liver **(C)**, and kidney **(D)** (*n* = 4 females per group). Fold change in expression of all genes is relative to 4-month-old tissue. ^∗^*P* < 0.05; ^∗∗^*P* < 0.01; ^∗∗∗^*P* < 0.001 (unpaired two-tailed Welch’s *t*-test with Dunnett’s multiple comparison). N.D. = Not detectable.

cDNA was synthesized from tissue-harvested total RNA using the SuperScript III kit (Invitrogen) per manufacturer’s protocol. All PCR reactions were carried out in a final volume of 10 μl and were performed in triplicate for each cDNA sample using Fast SYBR Green master mix (Applied Biosystems) on a StepOnePlus Real-Time PCR system (Applied Biosystems). The primer sequence for *p16^Ink4a^* is as previously published (Baker et al., 2016). All other primers utilized in this study are listed in **Table 1**. The ΔΔ*C*_t_ method was used to determine age-dependent gene expression changes, after normalizing the *C*_t_ values of each sample to at least two reference genes (*Actb*, *Gapdh*, *Hprt1*). Amplification of template was considered undetectable beyond a threshold of 35 cycles. A lack of detectable *p16^Ink4a^* expression in 4-month-old kidney prohibited an exact quantification of the magnitude of age-dependent upregulation, so to compensate for this lack, the level of *p16^Ink4a^* expression at 4 months was set equal to the threshold for detectable expression, and the minimum expression fold changes for the other age groups were calculated relative to this threshold (**Figure 1**). These calculations most likely underestimate the degree of increased *p16^Ink4a^* expression in the aged kidney. However, when calculated relative to both 12- and 24-month-old tissue, a significant upregulation was observed at 30 months (**Figure 1**). Statistical analysis was performed by two-tailed unpaired two-sample *t*-test with Welch’s correction and Dunnett’s multiple comparisons.

**Table 1:** Primer sequences used for quantitative RT-PCR.

Gene	Forward primer	Reverse primer
*Cdkn1a*	5′-GCAGATCCACAGCGATATCCA-3′	5′-AACAGGTCGGACATCACCAG-3′
*Cdkn2a*	5′-CCCAACGCCCCGAACT-3′	5′-GCAGAAGAGCTGCTACGTGAA-3′
*Il6*	5′-TGAGAAAAGAGTTGTGCAATGG-3′	5′-GGTACTCCAGAAGACCAGAGG-3′
*Il1a*	5′-AGGGAGTCAACTCATTGGCG-3′	5′-TGGCAGAACTGTAGTCTTCGT-3′
*Il1b*	5′-TGCCACCTTTTGACAGTGATG-3′	5′-TGATGTGCTGCTGCGAGATT-3′
*Timp1*	5′-CACACCAGAGCAGATACCATGA-3′	5′-GGGGAACCCATGAATTTAGCC-3′
*Mmp3*	5′-GTTGGAGAACATGGAGACTTTGT-3′	5′-CAAGTTCATGAGCAGCAACCA-3′
*Mmp12*	5′-TGCACTCTGCTGAAAGGAGTCT-3′	5′-GTCATTGGAATTCTGTCCTTTCCA-3′
*Cxcl1*	5′-ACCGAAGTCATAGCCACACTC-3′	5′-CTCCGTTACTTGGGGACACC-3′
*Cxcl2*	5′-CCCAGACAGAAGTCATAGCCAC-3′	5′-TGGTTCTTCCGTTGAGGGAC-3′
*Ccl8*	5′-CGGGTGCTGAAAAGCTACGA-3′	5′-TTGGTCTGGAAAACCACAGCTT
*Gapdh*	5′-CCCTTAAGAGGGATGCTGCC-3′	5′-TACGGCCAAATCCGTTCACA-3′
*Hprt*	5′-AGCAGTACAGCCCCAAAATG-3′	5′-ATCCAACAAAGTCTGGCCTGT-3′
*Actb*	5′-GTCCACACCCGCCACC-3′	5′-ACCCATTCCCACCATCACAC-3′


### Human Gene Expression Analysis

**FIGURE 2:**
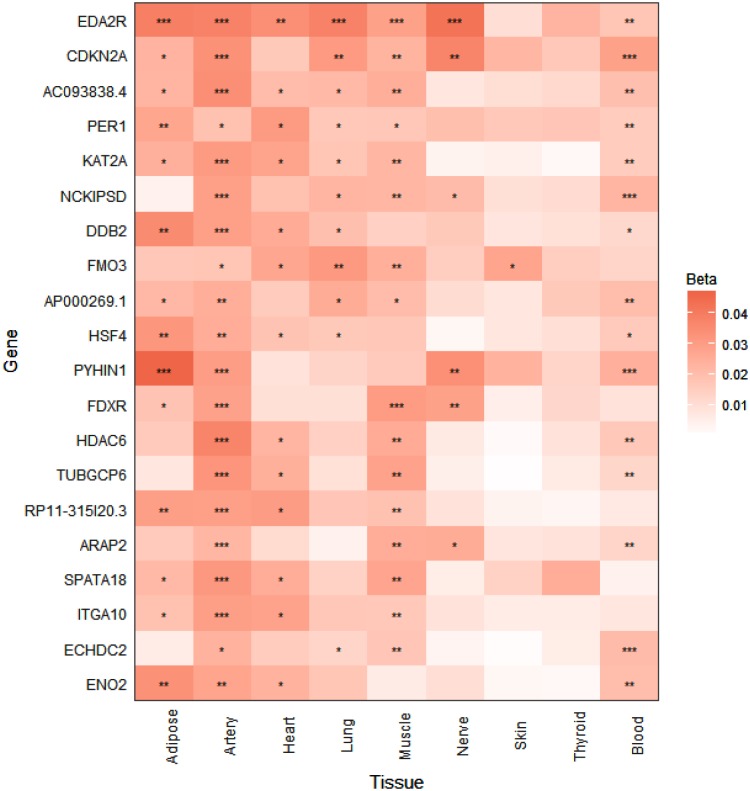
*p16^Ink4a^* expression is significantly elevated with age in multiple tissues in humans. Expression heatmap of the top 20 human genes showing increased expression with age across 9 tissues (subcutaneous adipose, tibial artery, left ventricle heart, lung, skeletal muscle, tibial nerve, skin from sun exposed lower leg, thyroid, and whole blood) analyzed by RNA-seq of the GTEx consortium. *CDKN2A* (*p16^Ink4a^*) exhibits significant increases in expression in 6 tissues with age. Genes are ranked from top to bottom according to number of tissues that show significant age-dependent upregulation. ^∗^*P* < 0.05; ^∗∗^*P* < 0.01; ^∗∗∗^*P* < 0.001 (FDR adjusted *p*-values). Beta represents the linear regression coefficient used to determine age-dependent expression change, with higher values indicating higher expression with age.

Data for the generation of the expression heatmap (**Figure 2**) came from the Genotype-Tissue Expression project (GTEx v3) RNA-seq analysis (Yang et al., 2015). The GTEx project provides whole transcriptome RNA-seq profiles for more than 40 tissues from hundreds of human donors of various ages, with the aim of increasing the understanding of how genetic variation affects the biology of common human diseases. In the Yang et al. (2015) study the authors utilized RNA-seq data from the nine GTEx tissues (subcutaneous adipose, tibial artery, left ventricle heart, lung, skeletal muscle, tibial nerve, skin from sun exposed lower leg, thyroid, and whole blood) that had sample sizes of greater than 80, which was then used to detect age-dependent gene expression changes according to a linear regression model that accounted for various confounding variables. For our analysis, age regression coefficients and FDR-adjusted *p*-values were extracted for all genes whose expression was found to increase with age across all nine GTEx tissues analyzed (Yang et al., 2015). This list of genes was then ranked and the heatmap was generated according to the number of tissues with FDR significant (<0.05) expression changes, as well as the magnitude of the corresponding adjusted *p*-values.

## Results

To determine the tissue- and age-dependent expression of senescence markers, we compiled a panel of 11 genes to be profiled, including 9 well-established SASP factors (Coppe et al., 2010a) – *Il6*, *Il1a*, *Il1b*, *Timp1*, *Mmp3*, *Mmp12*, *Cxcl1*, *Cxcl2*, and *Ccl8*, and two classical hallmarks of cellular senescence (Campisi and d’Adda di Fagagna, 2007) – *Cdkn2a* (*p16^Ink4a^*) and *Cdkn1a* (*p21^Cip1^*) (**Figure 1**). Four tissues were utilized for our analysis – hypothalamus, heart, liver, and kidney. The kidney (Sturmlechner et al., 2017), heart (Baker et al., 2016) and liver (Wang et al., 2009) have been shown to accumulate senescent cells with age and at the site of age-related pathology. We also chose the hypothalamus because it has been implicated in the regulation of the aging process (Zhang et al., 2017), as mediated through NF-κB signaling (Zhang et al., 2013), a major pathway involved in SASP factor induction (Malaquin et al., 2016).

We observed a dramatic difference in the tissue-specific expression profiles of the SASP factor/senescence marker gene panel with aging. In the hypothalamus, significant age-related expression changes were detected in *Il6*, *Il1b*, *Timp1*, *Mmp12*, *Cxcl1*, and *Cxcl2*, while in the heart only three genes, *Mmp12*, *Ccl8*, and *p16^Ink4a^* showed age-dependent changes in expression (**Figure 1**). In the liver, significant age-related expression changes were seen in *Il1b*, *Timp1*, *Mmp12*, *Cxcl2*, *p16^Ink4a^*, and *p21^Cip1^* (**Figure 1**). In kidney tissue, significant age-related expression changes were observed in *Il1a*, *Timp1*, *Mmp3*, *Mmp12*, *Cxcl1*, *Cxcl2*, *Ccl8*, and *p16^Ink4a^* (**Figure 1**). The only SASP factor that showed consistent age-dependent expression changes across all tissues analyzed was the matrix metalloproteinase *Mmp12*, which showed significantly increased expression in the tissues of 30-month-old mice, compared to 4-month-old mice (**Figure 1**). However, this increased expression was highly variable, with kidney expression increasing by an average of only 1.3-fold, while expression in liver showed a massive 26-fold average increase. In heart tissue, expression of *Mmp12* increased fivefold between 4 and 30 months (**Figure 1**). Intriguingly, tissue inhibitor of metalloproteinases 1 (*Timp1*) expression showed a 1.7-fold downregulation in kidney tissue between 4 and 30 months of age, which is consistent with *in vitro* senescence studies (Coppe et al., 2010a), but exhibited a >5-fold average upregulation between 4 and 30 months in liver tissue, and a >3-fold upregulation between 4 and 30 months in the hypothalamus (**Figure 1**). The expression of *Il6*, a major proinflammatory cytokine of the SASP (Coppe et al., 2008, 2010b), did not significantly increase with age in any tissue, although there was a trend toward increased expression observed (**Figure 1**). Surprisingly, there was a significant downregulation of *Il6* expression observed in hypothalamic tissue between 4 and 30 months. The classical biomarker of senescence, *p16^Ink4a^*, exhibited an age-dependent upregulation in the heart, liver, and kidney, but was below the limit of detection in the hypothalamus at all ages. For *p21^Cip1^*, no significant age-dependent alterations in expression were observed in the hypothalamus or the heart, but significant age-dependent upregulation was seen in both liver and kidney (**Figure 1**).

To investigate whether the tissue-specific and age-dependent changes in expression of senescence marker genes we observed in mice also occur during aging in humans, we examined human RNA-seq data from the GTEx project for age-associated changes in the expression profiles of our panel of senescence marker genes (Yang et al., 2015). When we ranked the GTEx tissue expression analysis by the number of tissues showing significantly increased expression with age, *CDKN2A* (*p16^Ink4a^*) was the found to be the second-most significantly elevated gene with age in multiple tissues in humans, including subcutaneous adipose, tibial artery, lung, skeletal muscle, tibial nerve, and whole blood (**Figure 2**). A significant age-dependent increase in the expression of *CDKN1A* (*p21^Cip1^)* was also found in the heart, lung, and whole blood (data not shown), while a significant age-dependent decrease in expression of *IL1B* was found in whole blood (data not shown). For *IL6*, *IL1A*, *TIMP1*, *MMP3*, *MMP12*, *CXCL1*, *CXCL2*, and *CCL8*, no significant age-dependent expression changes were found in any of the tissues analyzed (data not shown).

## Discussion

In the era of senolytics, it becomes imperative to develop robust biomarkers of senescence *in vivo* for preclinical trials, especially with several senolytics now nearing human clinical studies (Childs et al., 2017). As a first step, in this study we profiled the expression of a panel of known molecular markers of senescence in multiple tissues in mice at multiple ages, ranging from young (4 months) to very old (30 months). Because sex-specific differences were not a focus of this study, our analyses were conducted only on female CB6F1 hybrid mice, which have previously been reported to have an average lifespan of 30 months (Miller et al., 2005). This lifespan is considerably longer than the average lifespan of 25 months reported for female C57BL/6 mice (Turturro et al., 1999), one of the founder lines of CB6F1, presumably due to hybrid vigor. The results demonstrate that the secretory profiles and classical hallmarks of cellular senescence in aged tissues are highly variable and complex, suggesting that a systematic and concerted effort is needed to develop robust biomarkers of senescence for the identification, quantification, and monitoring of senescent cells *in vivo*.

Although none of the SASP factors and markers of senescence have been shown to be specific to the senescent state, even *in vitro* (Sharpless and Sherr, 2015), the wide diversity in tissue-specific profiles we observed was striking. Nevertheless, the matrix metalloproteinase *Mmp12* represents a robust SASP factor that showed consistent age-dependent increases in expression across all tissues analyzed in this study. It has been demonstrated that mice lacking *Mmp12* are protected from vascular injury, M2 macrophage accumulation, and perivascular heart fibrosis (Stawski et al., 2014). Together with our data, this finding suggests that *Mmp12* upregulation with age has a deleterious impact on heart function. In this study, we did not observe significant age-dependent upregulation of the prominent SASP cytokine *Il6* in any tissue, although an upward trend was observed that was consistent in magnitude with previous observations in the heart and kidney (Baker et al., 2016). This modest age-related upward trend could be explained by a previous report which demonstrated that senescent cell-secreted IL-6 acts in an autocrine manner, reinforcing the senescent state, rather than inducing senescence or promoting dysfunction in neighboring cells (Kuilman et al., 2008). In this case, the decreased expression of *Il6* with age we observed in the hypothalamus could be indicative of a lack or loss of senescent cells in that tissue with age. In support of this interpretation, *p16^Ink4a^* expression was non-detectable in the hypothalamus at any age. Taken together, these results suggest that some other age-related process results in the increased expression of the pro-inflammatory factors *Il1b*, *Mmp12*, *Cxcl1* and *Cxcl2* observed in the aged hypothalamus. Conversely, *p16^Ink4a^* expression was upregulated with age in all other tissues analyzed, consistent with previous reports (Krishnamurthy et al., 2004; Baker et al., 2016), and thus reinforcing the importance of *p16^Ink4a^* as a biomarker of tissue aging. Questions still remain, however, regarding the ultimate identity of the cells targeted for senolytic elimination in previous studies (Baker et al., 2011, 2016; Childs et al., 2016; Jeon et al., 2017), as it has been demonstrated repeatedly that *p16^Ink4a^* expression is not exclusive to senescent cells, and thus does not represent an unequivocal target for senolytic therapies (Sharpless and Sherr, 2015; Hall et al., 2016, 2017). Interestingly, however, *CDKN2A* (*p16^Ink4a^*) was one of the top human genes that exhibited elevated expression with age, in 6 out of 9 tissues, including subcutaneous adipose, tibial artery, lung, skeletal muscle, tibial nerve, and whole blood, as detected by RNA-seq analysis of the GTEx project (Yang et al., 2015). Thus, utilizing *p16^Ink4a^*-expressing cells as a biomarker of tissue aging and a target of senolytic therapies could prove to be an effective strategy in the future treatment of age-related diseases in humans.

Because we used tissue homogenates for the mRNA expression analysis, we were unable to determine if the senescence marker transcripts originated from cells native to the tissues under consideration, senescent or not, or from tissue-infiltrating cells of the immune system. Thus, our results should be interpreted with caution as general age-dependent trends of gene expression within these tissues. Since only female mice were used in our analyses, there is a possibility that the differential gene expression patterns we observed in mice could be sex-specific. However, to our knowledge, no prior study has reported any sex-specific expression differences of age-dependent senescence marker or SASP factor genes. To build on our findings, future work should expand the transcriptional analysis conducted in this study to a greater number of senescence marker genes, with concomitant measurements of protein abundances. More work is needed to fully characterize the expression profiles of SASP factors and senescence hallmarks in different tissue types at multiple ages in order to establish robust biomarkers of cellular senescence in mice.

## Author Contributions

AH, CT, and YS designed the experiments and analyzed the data. DH contributed mouse tissue. AH, CT, and AT performed all the experiments. All authors contributed to the final version of the manuscript.

## Conflict of Interest Statement

The authors declare that the research was conducted in the absence of any commercial or financial relationships that could be construed as a potential conflict of interest.
